# Outcomes of Pediatric Supracondylar Humerus Fracture Fixation in a District Hospital in Saudi Arabia: A Retrospective Study

**DOI:** 10.7759/cureus.83967

**Published:** 2025-05-12

**Authors:** Mansour M Aldhilan, Azeez O Tella

**Affiliations:** 1 Orthopedic Surgery, Al Rass General Hospital, Ministry of Health, Al Rass, SAU; 2 Orthopedics, Samtah General Hospital, Jazan, SAU

**Keywords:** closed reduction, district hospital, outcomes, pediatrics, supracondylar humerus fracture

## Abstract

Background

Supracondylar humerus fractures are among the most common pediatric fractures, and treatment can be challenging. Closed reduction and percutaneous pinning is the recommended treatment for displaced fractures. This study aimed to review the management of supracondylar humerus fractures at a district general hospital and to document our experience.

Materials and methods

We conducted a retrospective analysis of electronic medical records of pediatric patients who underwent surgical treatment of supracondylar humerus fractures treated over a 30-month period. Data collected included demographics, mechanism of injury, fracture type, modified Gartland classification, surgical management, clinical outcomes, and complications.

Results

A total of 36 patients met the inclusion criteria. The mean age was 5.2 ± 2.6 years (range 1-12 years), with a male-to-female ratio of 1.6:1. Extension-type injuries were observed in 35 patients (97.2%) and flexion-type in one patient (2.8%). According to the modified Gartland classification, extension-type fractures were distributed as follows: type II (34.3%), type III (60%), and type IV (5.7%). Most patients (91.7%) were treated with closed reduction and percutaneous pinning. The overall complication rate was 11.2%, with superficial surgical site infection being the most common (5.6%). One patient (2.8%) developed postoperative ulnar nerve palsy, attributed to medial pin placement. Based on Flynn's criteria, all patients achieved satisfactory outcomes at the final follow-up.

Conclusion

Our findings are consistent with those reported in the literature. Despite limited resources, closed reduction and percutaneous pinning remain the gold standard for managing displaced supracondylar humerus fractures, yielding satisfactory clinical outcomes and an acceptable complication profile.

## Introduction

Supracondylar humerus fractures are the most common type of elbow fracture in the pediatric population and typically require surgical fixation when displaced [1]. These injuries most commonly occur in children between five and seven years old [2] and can lead to several complications, including neurological, vascular, and compartment syndromes. Iatrogenic complications, particularly neurovascular injuries, may also occur. Loss of fracture reduction can also occur with surgical and nonsurgical treatments [3]. Supracondylar humerus fractures are classified into flexion and extension types. Extension-type fractures, which are more common, usually result from a fall onto an outstretched hand or hyperextended elbow [1,4]. The Gartland classification system is widely used for categorizing extension-type fractures. Type I fractures are non-displaced and typically managed conservatively. Type II fractures are displaced fractures but retain an intact posterior cortical hinge. Type III fractures are completely displaced without any hinges [5]. Type IV fractures, characterized by multidirectional instability in both flexion and extension, are usually diagnosed intraoperatively [6,7]. Flexion-type fractures are rare, accounting for approximately 2%-3% of supracondylar fractures [8]. These typically result from a direct fall onto the elbow, forcing it into hyperflexion. They are considered unstable and pose challenges in maintaining reduction and fixation [9]. For displaced supracondylar humerus fractures, the recommended treatment is closed reduction and percutaneous pinning [10]. Open reduction is indicated in cases involving vascular injuries or unsuccessful closed reduction [11]. Various pin configurations have been described in the literature, including lateral-only pinning or additional medial crossed pinning [12]. According to biomechanical principles, medial and lateral cross-pinning provides the greatest rotational stability [13]. Nonoperative management is reserved for non-displaced Type I fractures or selected Type II fractures where adequate closed reduction is achieved [14]. In Saudi Arabia, previous studies on supracondylar humerus fractures have focused on preoperative neuropraxia and its impact on surgical duration [15]. Another study examined the influence of surgical timing and surgeon experience on outcomes in a level I trauma center [16]. To our knowledge, no studies have evaluated the outcomes of pediatric supracondylar humerus fractures managed in district hospitals with limited resources. The aim of our study is to assess the outcomes of surgical treatment of these fractures in children and to share our experience managing them at a district general hospital in Saudi Arabia.

## Materials and methods

Patients

This retrospective study analyzed the medical records of pediatric patients aged 12 years or younger who underwent surgical fixation for supracondylar humerus fractures at Samtah General Hospital in Jazan, Saudi Arabia, between January 2021 and June 2023. Data collected included patient demographics, mechanism of injury, Gartland classification, time from presentation to surgery, follow-up duration, complications recorded, and timing of complications. Inclusion criteria were pediatric patients (≤12 years) with displaced supracondylar humerus fractures treated with either closed or open reduction and pinning. After applying exclusion criteria, only patients meeting the inclusion criteria were analyzed.

At the study institution, ethical approval is not required for retrospective analyses provided that patient data is properly deidentified. 

Preoperative evaluation

Each patient underwent an initial evaluation that included documentation of presenting complaints and the mechanism and circumstances of injury. This was followed by a comprehensive and meticulous clinical examination. Plain radiographs of the elbow, wrist, and shoulder were obtained in all cases to confirm the diagnosis and rule out associated fractures. Surgical intervention was rescheduled after obtaining anesthesia clearance or pediatric consultation when necessary.

Surgical intervention

Surgical intervention was performed under general anesthesia for all cases. In accordance with the hospital’s surgical prophylaxis protocol, all patients received cefazolin (30 mg/kg) within 30 minutes prior to the procedure. All procedures were conducted by specialists and/or consultants, including the two authors in most but not all cases. All patients had modified Gartland Type II fractures or higher. For closed reduction, the surgeon applied longitudinal traction to the forearm while the assistant provided counter-traction at the upper arm. Traction was maintained for about five minutes under radiographic control. Valgus/varus displacement was corrected using digital pressure. The elbow was gradually flexed to assess alignment on the lateral view. If reduction was deemed acceptable, percutaneous pinning was performed. If acceptable alignment could not be achieved after multiple closed reduction attempts, open reduction was performed using the posteromedial approach, with careful isolation of the ulnar nerve before pinning. Pin configurations included either all-lateral (parallel or divergent wires) or crossed pinning, with one K-wire inserted medially and one or two K-wires inserted laterally. To minimize the risk of ulnar nerve injury during closed procedures, two surface landmarks (the medial epicondyle and the olecranon) were marked with a sterile pen. A line connecting these points was assumed to represent the course of the ulnar nerve, and the medial K-wire was inserted anterior to the medial epicondyle under image intensifier guidance. A posterior splint was then applied with the elbow in 60-90 degrees of flexion.

Postoperative care

Patients who underwent closed reduction and pinning were discharged after 24 hours of observation, while those who had open reduction were discharged after 48 hours. Discharge criteria included a normal clinical examination and absence of patient complaints. Parents were instructed to closely monitor for signs such as abnormal swelling, increasing pain, and difficulty moving the fingers, for which they were advised to return to the emergency department if any of these symptoms occurred for further evaluation.

Follow-up

Follow-up visits were scheduled every two weeks, with plain radiographs taken at each visit. The pins were removed between four and six weeks, depending on the fracture's healing status. Follow-up evaluations focused on maintaining fracture reduction and assessing healing, with detailed documentation of any complications. Radiographic findings were recorded in each patient's medical file. After pin removal, physiotherapy was initiated, and patients were re-evaluated two weeks later. At this visit, Flynn’s criteria were assessed using a goniometer to measure clinical parameters, and the findings were documented.

Collection of data and statistical analysis

Data analysis was performed using IBM SPSS Statistics for Windows, Version 21.0 (Released 2012; IBM Corp., Armonk, NY, US). Categorical variables were presented as frequencies and percentages, and illustrated with charts where appropriate.

## Results

A total of 36 patients underwent surgical management during the study period. The mean age was 5.2 ± 2.6 years (range, 1-12 years). Among them, 22 were boys (61.1%) and 14 were girls (38.9%). Regarding the laterality of the fracture, 14 patients presented with right-sided fractures (38.9 %) and 22 with left-sided fractures (61.1%). The most frequent mechanism of injury was a ground-level fall (n = 32, 88.9%), followed by falls from height (n = 4, 11.1%). Extension-type supracondylar fractures were predominant, occurring in 35 patients (97.2%), while flexion-type was observed in only one patient (2.8%). Among the extension-type fractures, 12 were modified Gartland Type II (34.3%), 21 were Type III (60%), and 2 were Type IV (5.7%) (Table 1).

**Table 1:** Demographic and clinical characteristics of patient demographics

S no.	Age in years (mean 5.2 ± 2.6)	Gender (M:F = 1.6:1)	Side	Classification (modified Gartland)	Time from presentation to surgery in hours	Reduction technique	Follow-up period (mean 15.6 ± 7.2 weeks)	Flynn’s outcome rating
1	5	F	Right	Type III	48-72	Closed	12	Excellent
2	2	M	Right	Type III	24-48	Closed	12	Good
3	6	F	Left	Type III	<24	Closed	24	Fair
4	4	M	Right	Type IV	<24	Closed	12	Good
5	5	M	Left	Type III	24-48	Closed	24	Good
6	5	M	Right	Type II	24-48	Closed	12	Excellent
7	2	F	Right	Type III	24-48	Closed	12	Excellent
8	4	F	Left	Type II	<24	Closed	12	Excellent
9	2	M	Right	Type II	<24	Closed	12	Excellent
10	2	F	Right	Type IV	24-48	Open	16	Good
11	4	M	Right	Type II	24-48	Closed	12	Excellent
12	4	F	Left	Type III	<24	Closed	24	Fair
13	1	M	Left	Type III	24-48	Closed	12	Excellent
14	8	M	Left	Type III	<24	Closed	24	Good
15	7	M	Left	Type III	<24	Closed	12	Excellent
16	7	M	Left	Type II	48-72	Closed	12	Excellent
17	5	M	Left	Type II	<24	Closed	12	Excellent
18	11	M	Left	Type III	24-48	Open	24	Fair
19	5	M	Right	Type III	>72	Closed	18	Fair
20	6	F	Left	Type III	24-48	Closed	12	Excellent
21	8	M	Right	Type III	24-48	Closed	12	Excellent
22	2	F	Left	Type III	<24	Closed	12	Excellent
23	7	M	Left	Type III	<24	Closed	12	Excellent
24	7	F	Left	Type III	24-48	Closed	12	Excellent
25	3	F	Left	Type II	24-48	Closed	12	Excellent
26	12	M	Left	Type III	24-48	Open	24	Poor
27	6	M	Left	Type II	<24	Closed	12	Excellent
28	4	M	Right	Type III	<24	Closed	12	Excellent
29	6	F	Left	Type III	24-48	Closed	12	Excellent
30	8	M	Left	Type III	>72	Closed	24	Fair
31	2	F	Right	Type II	24-48	Closed	12	Excellent
32	9	M	Left	Type II	48-72	Closed	16	Excellent
33	8	F	Right	Type II	24-48	Closed	12	Excellent
34	5	F	Left	Type II	24-48	Closed	16	Excellent
35	3	M	Left	Type III	24-48	Closed	16	Good
36	4	M	Right	Type III	24-48	Closed	12	Excellent

**Figure 1:**
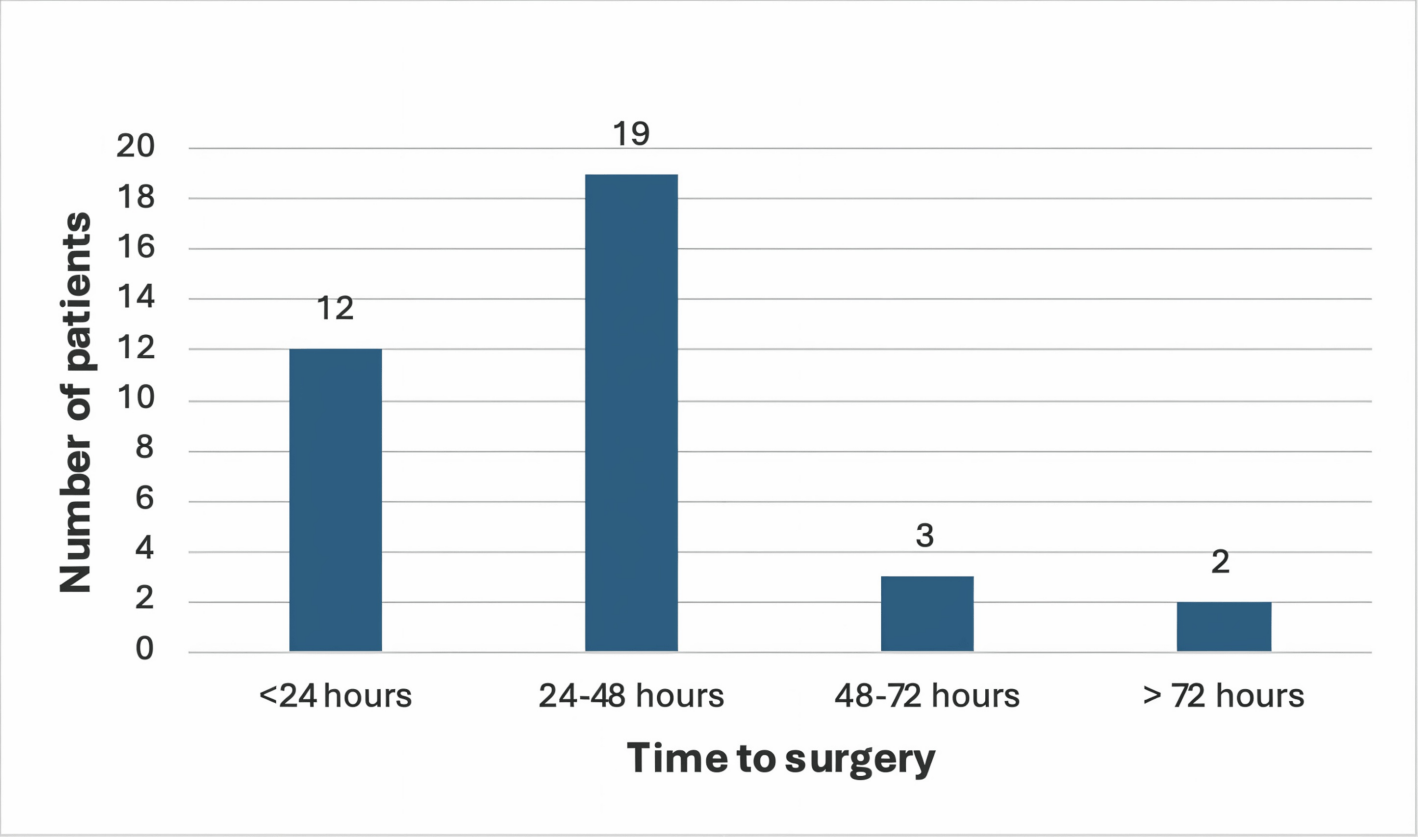
Duration between admission and surgery

Among the 36 patients in the study group, 12 (33.3%) underwent surgery within 24 hours of presentation, 19 (52.7%) between 24 and 48 hours, 3 (8.4%) between 48 and 72 hours, and 2 (5.6%) after 72 hours (Figure 1). 

**Figure 2:**
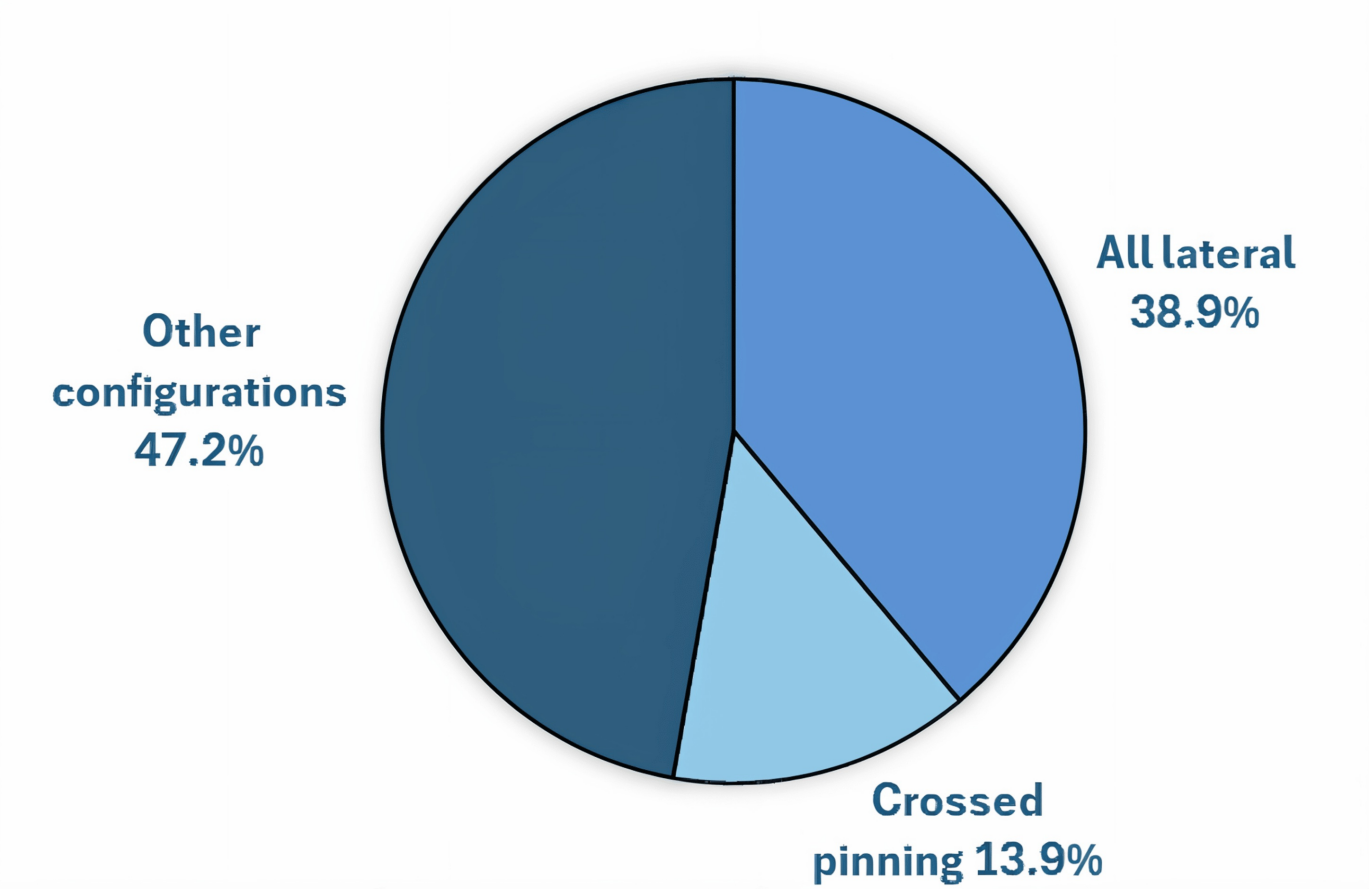
Pie chart showing the pinning configurations employed

Closed reduction and percutaneous pinning was performed in 33 patients (91.7%), while open reduction using the posteromedial approach was required in 3 patients (8.3%). Pinning configurations included all-lateral pins in 14 patients (38.9%) and crossed pins with one medial and one lateral wire in five patients (13.9%). Other configurations using multiple lateral and medial pins were employed in 17 patients (47.2%). Pinning configurations are illustrated in Figure 2. 

**Figure 3:**
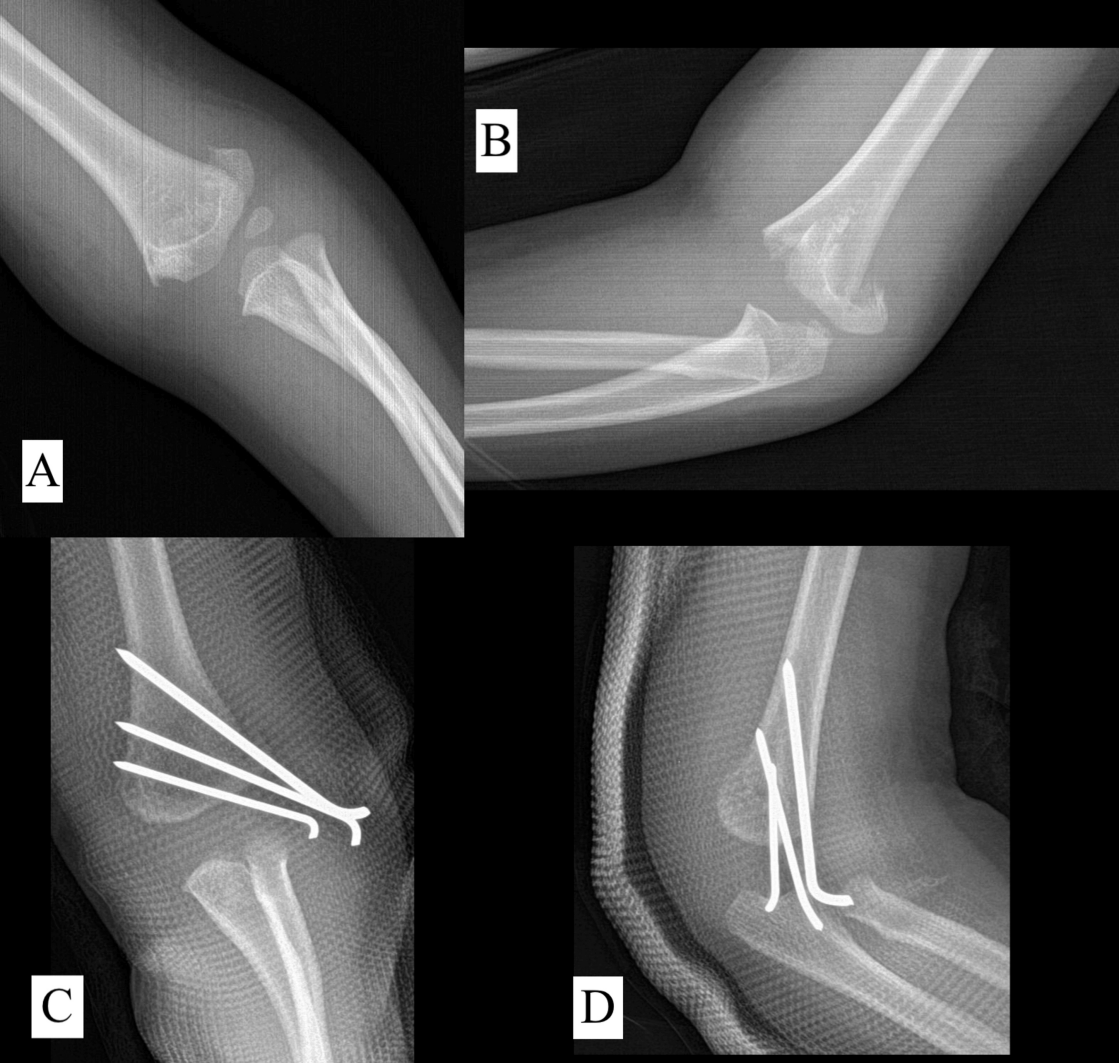
Radiograph of a three-year-old patient with a Type III supracondylar fracture (A) Preoperative AP view. (B) Preoperative lateral view. (C) Postoperative AP view showing all-lateral pin configuration. (D) Postoperative lateral view.

**Figure 4:**
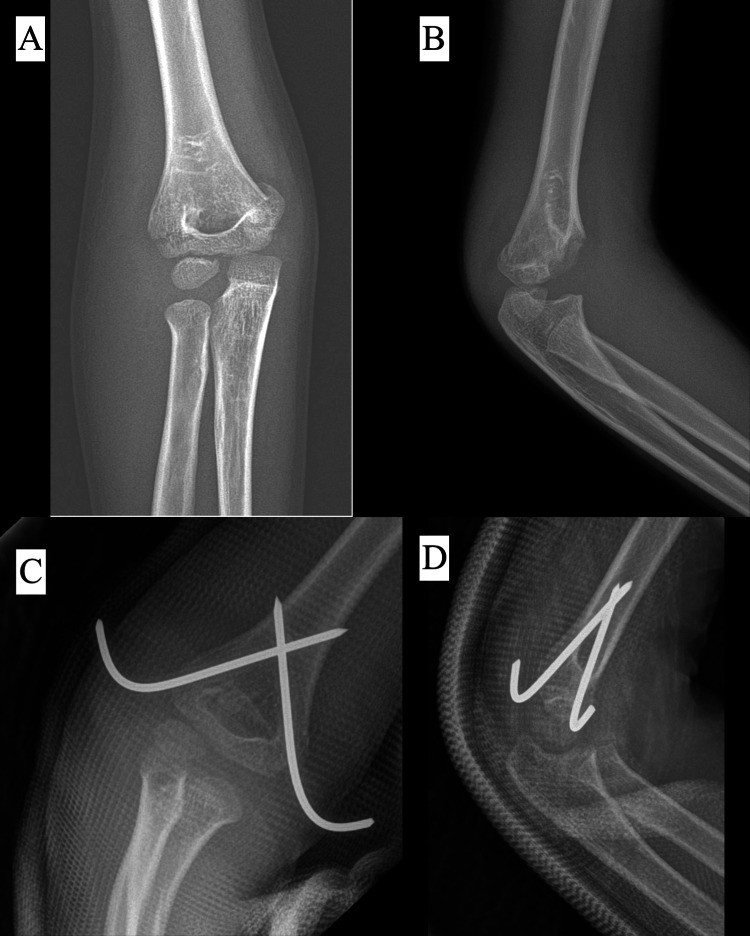
Radiograph of a five-year-old patient with a Type II supracondylar fracture (A) Preoperative AP view. (B) Preoperative lateral view. (C) Postoperative AP view showing crossed pin configuration. (D) Postoperative lateral view.

**Figure 5:**
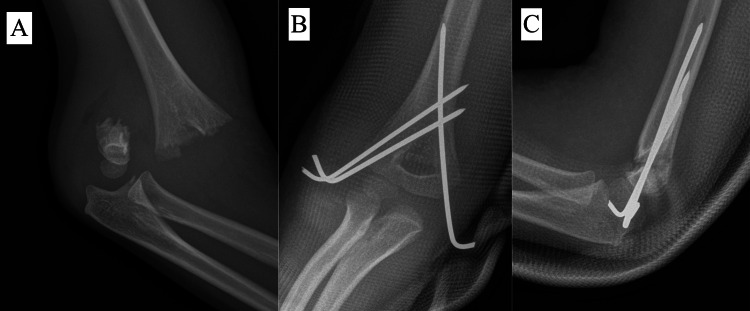
Radiograph of a four-year-old patient with Type IV supracondylar fracture (A) Preoperative radiograph. (B) Postoperative AP view showing other pin configurations. (C) Postoperative lateral view.

In our study, we used three different types of pinning configurations: all-lateral, comprising two or three lateral pins (Figure 3); crossed pinning, comprising one lateral and one medial pin (Figure 4); and other configurations of pinning, consisting of a combination of two lateral and one medial crossed pins (Figure 5).

The total duration of hospital stay was analyzed, with the majority of patients (72.2%) discharged within two days. The overall complication rate was 11.2%, including two cases of surgical site infection (5.6%), one case of iatrogenic ulnar nerve injury (2.8%), and one case of elbow stiffness (2.8%). The follow-up period ranged from 12 to 24 weeks, with a mean duration of 15.6 ± 7.2 weeks. Final functional outcomes were assessed using Flynn’s criteria for supracondylar humerus fractures (Table 2) [17].

**Table 2:** Flynn's criteria

Outcome	Rating	Functional factor: loss of range of motion	Cosmetic factor: loss of carrying angle
Satisfactory	Excellent	0-5⁰	0-5⁰
	Good	5-10⁰	5-10⁰
	Fair	10-15⁰	10-15⁰
Unsatisfactory	Poor	>15⁰	>15⁰

In our study, 23 patients (63.9%) achieved excellent outcomes, 8 (22.2%) had good outcomes, and 5 (13.9%) had fair outcomes. All the patients had satisfactory results at the final follow-up.

## Discussion

This retrospective study reviewed and analyzed the outcome of surgically treated displaced supracondylar humerus fractures at a district general hospital in Saudi Arabia.

The mean age of the study population was 5.2 ± 2.6 years, which is consistent with the widely reported epidemiology of pediatric supracondylar humerus fractures, which most commonly affect children aged 5-7 years.

In terms of the mechanism of injury, simple ground-level falls were the most common cause of fracture (88.9%) in our series. Similar findings were reported by Albrahim et al. [16], who conducted a retrospective study at a level I trauma center, identifying falls on an outstretched hand as the most common mechanism in 72.3% of cases.

According to most literature, extension-type injuries were the most common, occurring in 97%-98% of cases, while flexion-type injuries occurred in 2%-3%. In our series, extension-type injuries were seen in 97.2% of patients, with flexion-type injuries in 2.8%, consistent with these findings. Barr et al. [18] reported similar results, identifying 155 extension-type fractures (97%) and 4 flexion-type fractures (3%) in a cohort of 159 patients. In our study, Gartland Type III extension-type fractures were the most common subtype (60%). This contrasts with Barr et al. [18], who found Gartland Type I fractures to be most prevalent (46%), with Type III occurring in only 26% of cases. However, our findings align more closely with those of Albrahim et al. [16], who reported Gartland Type III fractures in 74.2% of their patients.

There are controversies regarding the optimal timing for displaced supracondylar humerus fracture fixation to minimize complications. Literature shows no differences in outcomes between early and delayed fixation. In our study, most patients (52.7%) underwent surgery between 24 and 48 hours after initial splinting. Suganuma et al. [19] and Okkaoglu et al. [20] categorized patients who underwent surgery within 12 hours as the early group and those treated after 12 hours as the late group. Both studies concluded that there was no correlation between early postoperative complications and delayed surgery for Type II or Type III fractures, indicating that the timing of surgery did not affect the quality of reduction and that surgery could be scheduled according to the surgeon's preference, as long as it is done within the first 24 hours.

In our study, 8.3% of patients (n = 3) required open reduction and pinning. Of these, two had modified Gartland Type III fractures, and one had a Type IV fracture. The primary reason for open reduction was the inability to achieve a satisfactory reduction using the closed method. In the series by DeFrancesco et al. [21], the rate of open reduction was 2.9% for Gartland Type III fractures and 22.2% for Gartland Type IV fractures. Similar to our study, the need for open reduction was due to the irreducibility of the fracture.

The choice of pinning configuration was determined intraoperatively based on the stability achieved, and the outcome of our study was not affected by the pinning configuration. Kocher et al. [22] and de Neira et al. [12] reported that all-lateral and crossed pinning configurations have comparable effectiveness in terms of rotational stability of the fractures.

In terms of complications, our study found one case of postoperative ulnar nerve palsy (2.8%), one case of elbow stiffness (2.8%), and two cases of surgical site infection (5.6%). The case of ulnar nerve palsy was related to the medial-sided pin insertion, which was repositioned via nerve exploration in another procedure. The patient did not have any long-term neurologic compromise. Barr et al. [18] reported two cases of postoperative ulnar nerve palsy (1.3%), which required nerve exploration, and the medial pin was the culprit in one of their reported cases. In a large series by Garg et al. [23] involving 1296 patients, they found 12% of patients had nerve palsy, of which 71% of nerve palsy were identified preoperatively and 29% postoperatively. The median nerve/anterior interosseus nerve (AIN) was the most frequently injured. Ulnar nerve injuries were the least frequent, occurring in only 16 patients, 9 of whom were diagnosed postoperatively. They concluded that the medial-sided pin did not increase the ulnar nerve injury rate, which contradicts our study.

In the series by Garg et al. [23], the infection rate was 2% (n = 18), with nine patients experiencing deep surgical site infection (1%) and the other nine (1%) having superficial infection. Our study found two patients with superficial surgical site infections (5.6%), and all cases of surgical site infection in our study were related to open reduction. The series by Barr et al. [18] showed 71 out of 1296 patients (8%) developed elbow stiffness, requiring outpatient physiotherapy. Our study found that one out of 36 patients (2.8%) developed elbow stiffness, managed successfully by physiotherapy consultation. In our study, the surgical site infection resolved with antibiotics and wound care; the case with iatrogenic ulna nerve injury had the surgery revised after five days, and the pin was repositioned. The main symptoms were tingling and abnormal sensitivity in the little finger, and all symptoms resolved with observation and physiotherapy after eight weeks. The patient with elbow stiffness reported slight improvement with physiotherapy and was referred to a higher hospital to continue treatment, based on our hospital’s guidelines.

The final outcomes assessed by Flynn's criteria in our study were satisfactory in all cases. We observed excellent outcomes in 63% (n = 23), good in 22.2% (n = 8), and fair in 13.9% (n = 5). The retrospective study by Krusche-Mandl et al. [24] reported satisfactory outcomes in 93.5% (n = 73) and unsatisfactory (poor) in 6.4% (n = 5), though they did not differentiate among excellent, good, or fair results. Another retrospective study by Davis et al. [25], which reviewed 87 operatively treated supracondylar humerus fractures, found excellent results in 56% (n = 19), good in 21% (n = 7), fair in 3% (n = 1), and poor in 21% (n = 7).

The limitations of our study include the relatively small sample size and the lack of comparable studies from district hospitals in Saudi Arabia. Additionally, the maximum follow-up period of 24 weeks may be considered short for assessing long-term outcomes.

## Conclusions

In conclusion, our study demonstrates that surgical treatment of displaced supracondylar humerus fractures in children is effective, with outcomes comparable to those reported in the broader literature. Further prospective level I and II studies conducted in district hospitals across Saudi Arabia are recommended to strengthen the evidence base and guide future clinical practice.
